# Genome-Wide Prediction of DNA Methylation Using DNA Composition and Sequence Complexity in Human

**DOI:** 10.3390/ijms18020420

**Published:** 2017-02-16

**Authors:** Chengchao Wu, Shixin Yao, Xinghao Li, Chujia Chen, Xuehai Hu

**Affiliations:** 1 College of Informatics, Agricultural Bioinformatics Key Laboratory of Hubei Province, Huazhong Agricultural University, Wuhan 430070, China; woo.kidd@gmail.com (C.W.); ccjljm@webmail.hzau.edu.cn (C.C.); 2 College of Science, Huazhong Agricultural University, Wuhan 430070, China; yaoshixin@webmail.hzau.edu.cn (S.Y.); lxh8930217@gmail.com (X.L.)

**Keywords:** DNA methylation, predicted model, sequence complexity

## Abstract

DNA methylation plays a significant role in transcriptional regulation by repressing activity. Change of the DNA methylation level is an important factor affecting the expression of target genes and downstream phenotypes. Because current experimental technologies can only assay a small proportion of CpG sites in the human genome, it is urgent to develop reliable computational models for predicting genome-wide DNA methylation. Here, we proposed a novel algorithm that accurately extracted sequence complexity features (seven features) and developed a support-vector-machine-based prediction model with integration of the reported DNA composition features (trinucleotide frequency and GC content, 65 features) by utilizing the methylation profiles of embryonic stem cells in human. The prediction results from 22 human chromosomes with size-varied windows showed that the 600-bp window achieved the best average accuracy of 94.7%. Moreover, comparisons with two existing methods further showed the superiority of our model, and cross-species predictions on mouse data also demonstrated that our model has certain generalization ability. Finally, a statistical test of the experimental data and the predicted data on functional regions annotated by ChromHMM found that six out of 10 regions were consistent, which implies reliable prediction of unassayed CpG sites. Accordingly, we believe that our novel model will be useful and reliable in predicting DNA methylation.

## 1. Introduction

Although the DNA sequence of the human genome, which carries genetic information, is almost invariant in various human cells, the epigenetic features of each cell show great differences, leading to distinguishable gene expression patterns and cell-type specificities [1]. Among these features (such as histone modifications mediating changes in chromatin conformation), DNA methylation is the best studied epigenetic modification [2,3]. In mammals, DNA methylation mainly occurs at CpG dinucleotide sites with an added methyl group to the fifth carbon of the cytosine residue [2,4,5,6,7,8]. In general, the patterns of DNA methylation are mediated by the main three enzymes, DNMT3 (for establishment), DNMT1 (for maintenance) and MBD4 (for demethylation) [3]. The human genome contains approximately 28 million CpG sites, 60%–80% of which are generally methylated [2], and the remaining unmethylated CpG sites are mostly located near promoter or exonic regions where GC content is much greater than 50%, which are usually called *CpG islands* (CGIs) [3,9].

The biological role of DNA methylation is to repress transcriptional activity. Different cell types show distinct methylation distributions across the genome, particularly in regulatory regions of cell-specific genes [2,3]. Notably, the average methylation levels in specific regions are consistent with other signals and modifications that are related to transcriptional regulation, such as transcription factor binding sites (TFBSs), DNase I hypersensitive sites (DHSs) and various histone modifications [1,9]. Recent studies have shown that hypomethylated regions were associated with promoters near transcriptional start sites (TSSs), whereas hypermethylated regions were considered as silenced regions. Interestingly, the low-methylated regions, in which average methylation levels are approximately 0.3, an intermediate status between hypomethylation and hypermethylation, were reported to be associated with distal regulatory regions, such as enhancers [9]. Therefore, DNA methylation is a key biomarker and plays a critical role in transcriptional regulation.

Considering the significance of DNA methylation, change of its level in specific regions is usually regarded as an important factor affecting the expressions of target genes and downstream phenotypes, such as embryonic development and tumorigenesis [10]. When compared to normal cells, aberrant average levels of DNA methylation in important regulatory regions (such as promoters) were linked with the altered expression profiles of cancer cells [11,12,13,14]. Interestingly, recent findings revealed that abnormal DNA methylation levels at distal regulatory regions (such as enhancers and super-enhancers) were closely related to gene dysregulation in cancer [14,15]. In summary, the average DNA methylation level in specific regions is an ideal biomarker of tumorigenesis, and detecting aberrant methylations in these regions is a promising approach for early diagnosis and classification of cancer [10].

Traditional methods for identifying DNA methylation mainly include whole-genome bisulfite sequencing (WGBS) [16], reduced representation bisulfite sequencing (RRBS) [17] and the methylation-microarray-based Illumina 450K BeadChip [18]. The 450K BeadChip uses microarray-based technology to assay approximately 0.4–0.6 million preselected CpG sites. In contrast, WGBS is a standard method in whole-genome sequencing at base resolution with approximately 1–18 million CpG sites captured [19]. Additionally, RRBS is an efficient technique designed for enriching the regions with a high CpG content, leading to high-efficiency and saving compared with WGBS. The total coverage of RRBS is roughly equivalent to that of WGBS, and the number of CpGs captured per sample genome by RRBS ranged from 0.5 million to 13 million [19]. However, there are 28 million CpG sites in the whole human genome, only 10%–50% of which are covered by WGBS or RRBS. Why it is difficult to assay the majority of CpGs in the whole genome even by the high-throughput techniques? Generally, there are many experimental difficulties hampering the analysis of the methylation status within this “hidden” fraction of CpG sites, such as copy-number variation bias, incomplete bisulfite conversion bias, bisulfite PCR bias, GC content bias and CpG density bias [19]. For RRBS or WGBS experiments, still two coverage limitations (incomplete bisulfite conversion bias and bisulfite PCR bias) could happen [19,20,21], which are the reasons for their low coverages. Therefore, more quantitative methods are urgently demanded to predict the methylation status of the remaining unassayed CpG sites of the human genome.

Computational approaches are alternative methods to identify DNA methylation status. Actually, a number of computational methods had been developed to predict DNA methylation status with the rapid developments of bioinformatics and machine learning approaches, which usually contain three vital steps—data collection, feature extraction and a classification algorithm [22]. Similarly, many efforts have been made for predicting the functional sites in proteins (such as cysteine S-nitrosylation sites [23,24,25,26,27], protein methylation sites [28], hydroxyproline and hydroxylysine in proteins [29], lysine ubiquitination sites [30], lysine succinylation sites [31]. For more details, please refer to two recent reviews [32,33].

Due to the restrictions of experimental data (such as microarray), some early methods limited their attentions on specific genomic regions (such as CGIs) and obtained satisfactory prediction accuracies (with accuracy >90%) [34,35,36,37]. In contrast, when turning to more common regions predicted using RRBS and WGBS data, the prediction accuracies clearly decreased to 75%–89% [38]. This is because when we focused on the prediction in CGI regions, GC contents of positive samples are high, whereas GC contents of negative samples are lower. Thus, it is easy to distinguish the methylated CpGs and unmethylated ones only by GC content feature. However, when turning to more common regions, the predictive role of GC content will not be as significant as that of CGI regions and the corresponding prediction accuracies will decrease. In these situations, more complex features are needed for better predictions. 

Regarding feature extraction, various methods have been employed to formulate methylation status, including: DNA composition [34,36,39,40], pseudo trinucleotide composition (PseTNC) [41,42,43,44,45], predicted DNA structure [34,46], single nucleotide polymorphisms (SNPs) [34], TFBSs [34,46], histone modifications [36,46], neighboring CpG site methylation status and distance [46]. It is worth noting that a powerful web-server called “Pse-in-One” has been used to extract various features from DNA or protein sequences [47].

Particularly, Das et al. analyzed GC content and Alu elements features for 800 bp regions centered on CpG sites using DNA methylation data of human brain, and developed classifiers with accuracy of 86% [40]. Moreover, Bock et al. employed 1184 DNA attributes for discriminating between CpG islands that are prone to methylation from those that remain unmethylated using CpG island methylation data on human Chromosome 21, and they found that certain sequence patterns, specific DNA repeats and a particular DNA structure played significant roles in the prediction of DNA methylation status [34]. In the same year, Fang et al. [39] used nucleotide sequence features including GC content, CpG ratio, TpG content and Alu distribution, as well as TFBSs features to predict DNA methylation status on human brain dataset. They tested four different window sizes (200 bp, 300 bp, 400 bp, and 500 bp) and found 400 bp is a better choice for prediction, with accuracy of 85%. Recently, Liu et al. invented a novel method called “pseudo trinucleotide composition (PseTNC)” which can both capture the local or short-range sequence order effects and the global or long-range effects of DNA sequences for predicting the methylation status of DNA fragments using 41-bp window centered on CpG sites [41]. Notably, Zhang et al. [46] integrated four groups of features, including neighboring features, genomic position, DNA sequence properties and *cis*-regulatory elements, to predict DNA methylation status using the whole blood sample dataset. As a result, 400-bp window was also found to be the best size for prediction and DHSs as well as GC content were found to be the most predictive features. In addition, Wang et al. combined “PseTNC” and chromatin interaction features (Hi-C features) to predict methylation states using the methylation datasets taken from two cell lines (GM12878 and K562). A series of window sizes (500–1000) were tested to search the best one, and 600 bp was found to be the appropriate choice [48].

These methods were mainly developed based on some common machine-learning algorithms: support vector machine (SVM) [34,35,36,38,40,49], random forest (RF) [37,46], naive Bayes (NB) and stacked denoising autoencoders (SDA) [48]. One can refer to the textbook written by James et al. [50] for more details of these algorithms and their implementations in R. The majority of these studies used SVM as the classifier due to its powerful classification ability and universality for various data types. Notably, some research showed the superiority of RF compared with SVM [46], and the SDA method from deep learning field emerged for prediction of DNA methylation [48].

The methods above achieved remarkable results, but there were still some defects among them: (a) most of them could not perform predictions at the whole genome-wide level because the data they used were specific fragments of the genome (such as the 450K BeadChip); (b) although some of them developed their classifiers based on RRBS and WGBS and achieved satisfactory results [46], obtaining important features for prediction (such as neighboring CpG site methylation status and DHSs) is difficult and would require comprehensive and expensive epigenetic experiments; and (c) most of them did not test the validities of their predicting models on unassayed CpG sites.

In this work, we introduced a novel computational algorithm called “sequence complexity”, together with DNA composition (72 features in total) to predict the DNA methylation status of CpG sites in the whole human genome. Our prediction method has the following advantages compared with current classifiers: (a) a group of novel features called “sequence complexity” were developed, and subsequent analysis confirmed that these new features played significant roles for predictions; (b) by integrating the fundamental features (DNA composition), the prediction model achieved satisfactory results; (c) all the features we used were only extracted from the primary DNA sequence of the human genome without additional experiments, and comparisons with previous works showed the superiority of our method; and (d) a statistical test of the experimental data and the predicted data on functional regions annotated by ChromHMM found that six out of 10 regions were consistent, which implies reliable prediction of unassayed CpG sites. Thus, we believe that our novel model will be useful and reliable in predicting DNA methylation.

As illustrated by many recently published papers [51,52,53,54,55,56,57], to establish a powerful predictor for a biological problem, one should obey the following five steps: (a) build or choose a benchmark dataset to train and test the predictor; (b) transform the raw biological sequences into mathematical feature vectors that can truly extract their intrinsic features from the target to be predicted; (c) employ or create a powerful algorithm to operate the prediction; (d) accurately use cross-validation tests to impersonally evaluate the predicting ability of the predictor; and (e) build a user-friendly web-server to make their dataset and predictor publicly available. Next, the current study will be organized following these steps one-by-one.

## 2. Results

### 2.1. DNA Methylation Dataset and Data Preprocessing

In this work, we developed a SVM-based model to predict the DNA methylation status of CpG sites in the human genome. In this work, Homo sapiens embryonic stem cell methylation profiles that were measured by RRBS [58] were downloaded from the NCBI Gene Expression Omnibus (GEO, GSE49828). After downloading, eight important cell stages (MII oocytes; zygotes; 2-cell, 4-cell, and 8-cell embryos; morulae; ICM of blastocysts; and post-implantation embryos) out of 12 were selected for analysis. There are 27,762,346 methylation sites, 4,476,329 of which are CpG sites. Embryonic stem cells are suitable for comprehensively studying DNA methylation status for two reasons. One is the large amount of number of assayed CpG sites (more than ten million CpG sites in this research) [58], because our machine-learning system requires larger samples for reducing the false positive rate. The other is the strategy we used for selecting positive and negative samples, which are stably methylated or unmethylated during all eight stages of embryonic stem cells. Whereas the adult cells display a cell-specificity patterns of DNA methylation.

Specifically, given a CpG site in a fixed stage, its methylation level is quantified by a variable *β*, which represents the number of methylated reads divided by the sum of both methylated and unmethylated reads at the same positions of the reference genome. Therefore, the term *β* of each CpG site ranges from zero (unmethylated) to one (fully methylated). Here, we adopted a similar strategy to that of Wang et al. [48] for choosing positive and negative samples: the intersection of CpG dinucleotide sites with methylation levels *β* ≥ 0.7 during all eight stages were labeled positive, whereas the intersection of CpG dinucleotide sites with methylation levels *β* ≤ 0.01 during all 8 stages were labeled negative. Through this procedure, 139,931 methylated CpG sites (139,931/4,476,329 = 3.13%) and 536,183 (536,183/4,476,329 = 11.98%) unmethylated CpG sites were obtained for further work.

To investigate the appropriate window size for prediction, for each CpG site, a series of DNA sequence window sizes (100 to 1000 bp) with the CpG site as its center, were generated using the HG19 reference genome. For comparable computations and so that different window sizes have the same number of positive and negative samples, we focused on the DNA sequence window size of 1000 bp for all CpG sites and the other window sizes were just the subsets of them. Through this procedure, a positive dataset consisting of 139,931 1000-bp windows was obtained, and a negative dataset consisting of 536,183 1000-bp windows was also obtained in the same manner, which constitutes our original dataset.

To reduce the homology bias of prediction, a redundancy reduction procedure was performed on the above original dataset using the CD-HIT program [59] (http://www.bioinformatics.org/cd-hit/), and a cutoff threshold of 0.8 was imposed to exclude those DNA sequences that have 80% or greater sequence identity to any other in a same subset. Based on this pre-processing procedure, the positive and negative datasets were determined (104,876 + 159,090), and are shown in Table 1.

### 2.2. DNA Methylation Pattern

**Figure 1:**
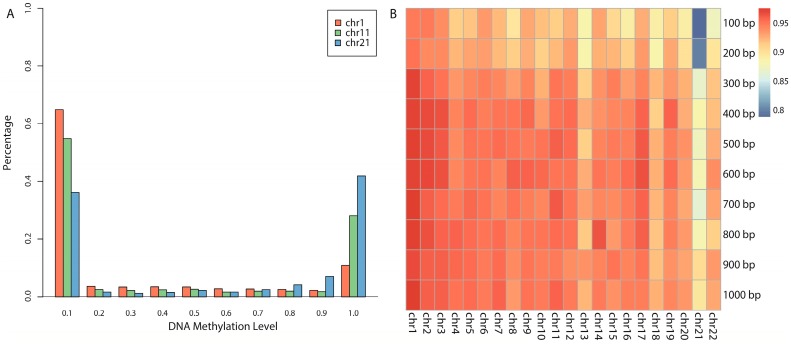
DNA methylation patterns and the heat map of overall prediction accuracies: (**A**) bimodal distributions of DNA methylation patterns; and (**B**) the heat map of prediction results for combinations of different chromosomes and different window sizes.

To better understand the DNA methylation profiles, the distribution of DNA methylation level *β* in the whole human genome is shown in Figure 1A. For simplicity, we chose three representative chromosomes (chr1, chr11, and chr21) by size and gene number for exhibition. As in previous studies [2,3,46,48], the DNA methylation level of each chromosome all showed a bimodal landscape, i.e., most CpG sites either have fully methylated status (*β* ≥ 0.9) or have unmethylated status (*β* ≤ 0.1), and few have intermediate methylated status (0.1 < *β* < 0.9). Notably, the three representative chromosomes generally showed bimodal distributions, but their distributions still showed some differences (Figure 1A). For example, chromosome 1 has more unmethylated CpG sites, whereas chromosome 21 has more fully methylated CpG sites. This explains why different chromosomes present different prediction accuracies in the following analysis because changing in distribution will result in changing of prediction result by the basic principle of statistical learning theory.

### 2.3. Overview of Binary Methylation Status Prediction

Here, we focused on binary methylation status prediction, which asserted that the methylation status of each CpG site was encoded as a binary variable (1 for fully methylated sites, 0 for unmethylated sites). Several previous studies have all found that DNA methylation status was strongly correlated with local DNA sequence [34,35,36,37,38,39,40,41,46,48]. To investigate which windows are most predictive for methylation, we tested our prediction approach with ten different window sizes (100–1000 bp).

Concerning feature extraction, 72 features were used to formulate DNA fragment sequences with different lengths, which included two groups of features (for details, see Materials and Methods):

1. DNA composition (DC, trinucleotide frequency and GC content, 65 features); and
2. Sequence complexity (SC, 7 features).

**Figure 2:**
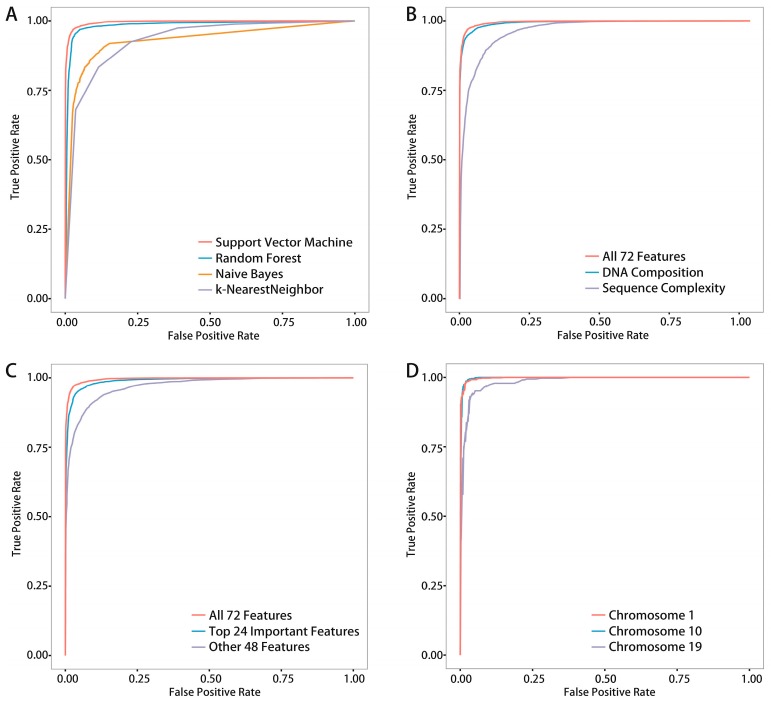
Receive Operating Characteristic (ROC) curves of different comparisons: (**A**) ROC curves of comparisons between two groups of features using 10-fold cross-validation; (**B**) ROC curves of comparisons between the top 24 important features and remaining 48 features using independent testing; (**C**) ROC curves of comparisons between four common classifiers using independent testing; and (**D**) ROC curves of three mouse chromosome predictions.

For the classifier, SVM was chosen by comparisons between the other three common classifiers (Figure 2A). For the evaluation method, 10-fold cross-validation (see Materials and Methods) was chosen for testing the prediction performance of our method.

An overview of prediction results was exhibited in the form of a heat map (Figure 1B), in which each grid represented a corresponding chromosome and window size used for prediction (for detailed values, see Table S1). The closer to red, the better the prediction result was in that grid, whereas, the closer to blue, the worse the result was.

Firstly, we observed that all chromosomes with all window sizes achieved prediction accuracies ≥0.8. Secondly, if we focused on each column (representing each chromosome), the best results are all above 0.9 even in chromosome 21 (chromosome 21 with a 900-bp window, 0.9022, see Table S1). These results imply that our approach is a powerful predictor for DNA methylation. Notably, if we focus on each row, the 600-bp window shows consistent predictive power, and 40% of chromosomes (9 out of 22) achieve the best accuracies with 600-bp window. Even in the remaining chromosomes, the prediction differences between 600-bp window and the corresponding window in which the best accuracy was achieved were not significant (*T*-test, *p* value = 0.3521). This leads us to confirm that 600 bp is the most appropriate window for DNA methylation prediction. Hereafter, we only focused on 600-bp windows with the CpG site as its center in the rest of paper.

### 2.4. Comparison with Different Classifiers

To test which classifier would achieve the best prediction result and to explain why we chose SVM as the classifier in this work, a detailed comparison with other classifiers was performed. Here, three frequently used classifiers, Random Forest (RF), Naive Bayes (NB) and K-Nearest Neighbors (KNN), were chosen to compare with SVM (see Table 2) and their respective ROC (Receiver Operating Characteristic) curves in Figure 2A.

A smaller subset of the whole dataset was constructed for subsequent in-depth analyses due to the computation requirements for large samples. This subset contains a training sample-set (T-set) with 5000 positive samples and 5000 negative samples (randomly selected from all chromosomes) and an independent testing sample set (IT-set), which has no overlaps with the T-set with another 5000 positive samples and 5000 negative samples. As a result, SVM achieved the highest AUC (Area Under Curve) of 0.996% and ACC (accuracy) of 97.1%, and RF also performed well with the similar prediction with AUC of 0.984% and ACC of 95.9%. The other two classifiers could not achieve satisfactory prediction results compared with SVM.

### 2.5. Feature Importance

In Figure 1B, we found that 72 features performed well for our prediction. However, each feature may not contribute equally to prediction. Subsequently, we used the T-set and IT-set with SVM to discover the importance of each feature.

We examined the contributions of two groups by plotting the ROC (Receiver Operating Characteristic) curves of each feature group (see Figure 2B and Table 3) and listing their evaluation indexes (see Table 3). As a result, it was found that fusional method with all 72 features achieved the best prediction accuracy of 0.971, and DC, SC achieved accuracies of 0.946 and 0.910 respectively. Notably, although DC achieved ACC of 0.946 which is greater than 0.910 achieved by SC, average contribution of each feature of SC is 0.910/7 = 0.13 which is nearly tenfold greater than that of DC (0.946/65 = 0.015). These results reveal that DC is the fundamental group of features and SC is an important complementary group of features for predicting DNA methylation.

**Figure 3:**
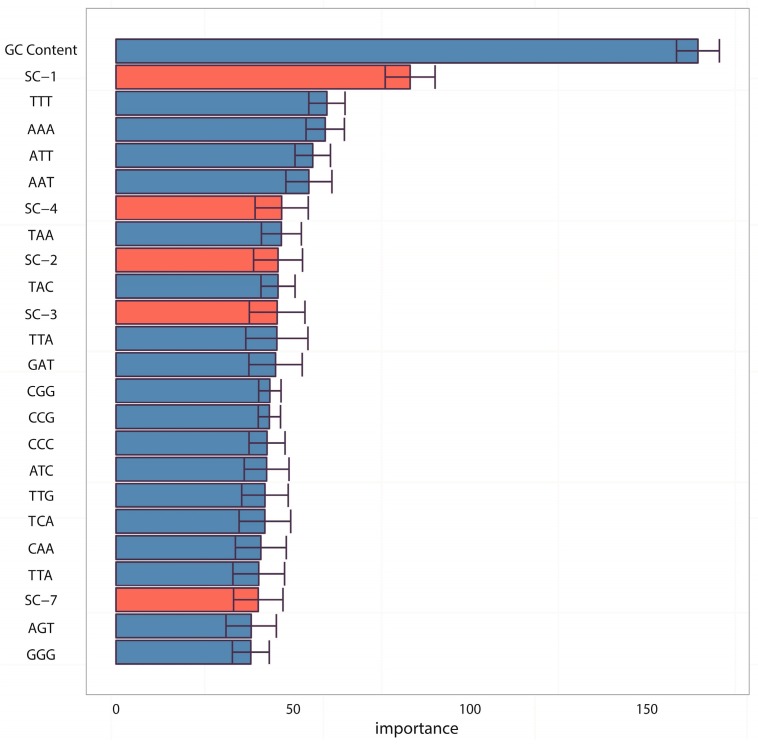
Top 24 important features by normalized regression coefficients in linear kernel SVM. The importance of the features was obtained by resampling statistics and the corresponding error bars of the top 24 features are represented. The colors represent different groups of features: DNA composition is blue, and sequence complexity is red.

Furthermore, we also evaluated the contribution of each feature by its feature importance in the SVM classifier, which was computed as the normalized regression coefficients of each feature in the linear kernel SVM. The feature importance map of the top 24 features according to their ranks is shown in Figure 3, in which GC content was shown to be the most important and five SC features (for more details of SC features, please refer to Material and Methods) were found in the top 24 features. Among them, SC-1 feature (the first feature of SC features, that is the fourth point of complexity function for the 600-bp window) achieved the second rank and SC-2 (the second feature of SC features, that is the fifth point of complexity function for the 600-bp window) was ranked in 9th, which was consistent with a previous work [60] that found the preceding points of entropy points (SC-2 here for 600 bp) were very important for describing complexity information of DNA sequence. In this work, we further found that the subsequent points of entropy points (SC-3 and SC-4, ranked in 11th and 7th, respectively) also provided additional information for prediction. Finally, traditional DNA motif features reported in previous works [40,48], such as TTT, AAA, CGG, CCG, etc., were also proven to be of high importance.

To further show that the top 24 important features are significant, the remaining 48 (72 − 24 = 48) features were used to perform prediction and the corresponding predicting accuracy was only 90.7%, whereas the top 24 important features achieved a 95.8% prediction accuracy with only half the features (Figure 2C and Table 4).

### 2.6. Comparison with Other Existing Methods

To demonstrate the superiority of our method, a detailed comparison with existing methods was performed. A number of prediction methods were developed for identifying the methylation status of CpG sites, but not all methods are suitable for comparison, because some were constructed more than ten years ago and others did not make their dataset publicly available. Here, we chose two recently published existing methods with accessible datasets for comparison, **iDNA-Methyl** [41] and **DeepMethyl** [48]. 

For comparison with **iDNA-Methyl**, we downloaded its dataset (787 methylated samples and 1639 unmethylated samples) from their website (http://www.jci-bioinfo.cn/iDNA-Methyl) and then computed our 72 dimensional features. The detailed comparison result in based on evaluation indexes is shown in Table 5, from which we found that the overall prediction accuracy (ACC) of our method was 78.62%, outperforming **iDNA-Methyl**, which had an ACC of 77.49%. In addition, the sensitivity and specificity of our method are 78.68% and 78.56%, respectively, which are more balanced than those of **iDNA-Methyl** (61.25% and 90.33%, respectively). This implies that the prediction results are reliable for identifying both methylated samples and unmethylated ones, whereas **iDNA-Methyl** only accurately identified 60% of methylated samples, though it can precisely predict 90% of unmethylated samples.

For another predictor, **DeepMethyl**, we downloaded the methylation data for cell lines GM12878 (a B-lymphocyte cell line from a normal female) and K562 (an immortalized cell line from a female patient with chronic myelogenous leukemia) from the ENCODE project (http://hgdownload.cse.ucsc.edu/goldenPath/hg19/encodeDCC/wgEncodeHaibMethylRrbs/, the GEO accession numbers and detailed number of positive and negative samples can be found in Table 1). For the results, satisfactory prediction accuracies were achieved for the GM12878 cell line (Table S2), and all 22 chromosomes had accuracies greater than 96% with an average accuracy of 97.93%. For K562 cell line, the prediction results were also very good and the average accuracy was 97.18% (Table S2). For comparison with **DeepMethyl**, we listed sectional comparisons because only those results appearing in Table 6 can be found in the **DeepMethyl** paper [48], and all the prediction results termed as ACC are displayed in Table S2. Interestingly, for GM12878 cell line, we found that our method achieved an ACC of 98.4%, outperforming **DeepMethyl**, which had an ACC of 90.0% in larger chromosome 1, and also outperforming it 98.3% compared to 94.2% in smaller chromosome 22. Similarly, our method outperformed **DeepMethyl** for K562 cell line. In addition, another contribution of this work is to provide comprehensive prediction results for all chromosomes in both the GM12878 and K562 cell lines, whereas **DeepMethyl** provided incomplete information in their paper.

### 2.7. Cross-Species Prediction

To show that our method has certain generalization ability, we chose the mouse genome (another mammalian animal) for cross-species prediction. Fortunately, a similar recently-published paper studied mouse embryonic stem cell methylation status for the same eight stages [61]. We downloaded the corresponding dataset from the NCBI Gene Expression Omnibus (GEO, GSE56697, see Table 1). Subsequently, each sample was processed into a 600-bp DNA fragment with a methylated CpG site (positive sample) or a unmethylated CpG site (negative sample) in its center using the mouse reference genome mm10.

DNA methylation in mouse was predicted with very high accuracy, in fact even higher that in human (Table S3). The average accuracy of all 19 chromosomes was 97.37%, and the average AUC value was 0.9955. For convenience, we listed the important evaluation indexes of each chromosome in Table S3 and generated the ROC curves of three chromosomes out of 19 chromosomes for an intuitive display (Figure 2D and Table 7).

### 2.8. Prediction of DNA Methylation Profiles across the Whole Human Genome

Although the prediction of methylation status of CpG sites is important, accurate prediction of the DNA methylation profile of specific genomic region (the average methylation level of all CpG sites within this region) is more practical when considering their biological functions. Therefore, we applied a robust strategy to show the practicability of our model by comparing the differences between the experimental methylation profiles and predicted methylation profiles (predicted by our trained model) of some important functional regions of the human genome. ChromHMM [62] is a commonly used method that systematically segments the human genome into different functional regions according to patterns in the presence or absence of multiple chromatin marks, such as the ChIP-Seq data of various histone modifications. Significantly, many published studies have adopted ChromHMM to display their results, including the famous Epigenome Roadmap Project [1].

**Figure 4:**
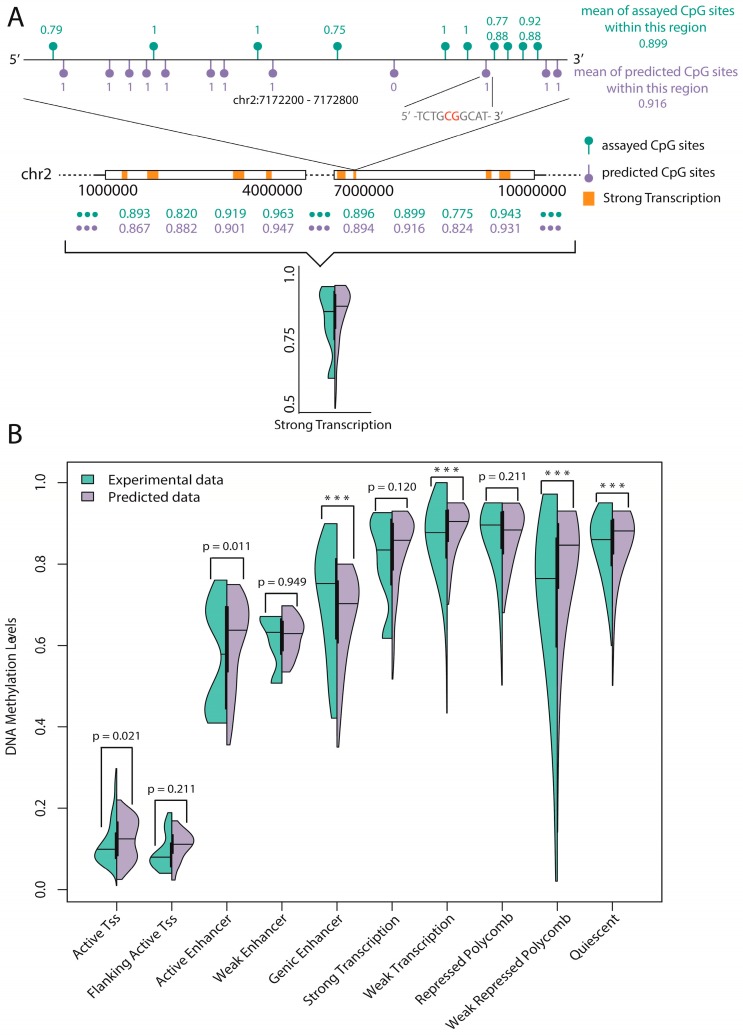
Statistical tests of the experimental data and the predicted data on 10 functional regions: (**A**) the computing procedure for one of 10 regions (Strong Transcription); and (**B**) the semi-violin plots show the distributions of average DNA methylation levels on 10 functional genomic regions. The *p* values of the six regions confirmed to be statistically consistent are labeled, and *** represents *p* < 0.0001.

Here, we also applied the annotation information provided by ChromHMM to show our results. For each sample within a specific region, we chose only the suitable DNA fragments that contained more than 10 “assayed” CpG sites (=those for which DNA methylation data are available) and 10 “unassayed” CpG sites (=those for which there is no experimental data for DNA methylation), and then calculated the average methylation level of such a sample (Figure 4A). Thus, only 10 regions (active transcription start site, flanking active transcription start site, active enhancer, weak enhancer, genic enhancer, strong transcription region, weak transcription region, repressed Polycomb state, weak repressed Polycomb state and quiescent state) out of 18 were selected to test the prediction because the sample numbers of unselected regions were not large enough to perform statistical tests (less than 1000).

As a result, two semi-violin plots that clearly show the differences between the experimental data and predicted data were shown in Figure 4B. Furthermore, a Wilcox test was employed to test the significant differences between the experimental and predicted results for all 10 regions, and 6 regions including active transcription start site, flanking active transcription start site, active enhancer, weak enhancer, strong transcription region and repressed Polycomb state were confirmed to be not statistically dissimilar (*p*-value > 0.01). The remaining regions, though statistically significant, did not appear to be discrepant (Figure 4B). Thus, our model has the ability to accurately predict the methylation profiles of important regions across the whole human genome, which also reveals the robustness of our work.

## 3. Discussion

We proposed a novel algorithm that could accurately extract the sequence complexity features of DNA methylation status, and developed a SVM-based prediction model by integrating DNA composition features based on human embryonic stem cell methylation profiles. Different window sizes (100–1000 bp) and different chromosomes were combined pairwise to display the overall prediction results, and 600-bp windows whose center are methylated CpG sites were found to achieve the best accuracies. 

In the analyses of feature importance, the feature group of DNA composition was found to be the fundamental features and the feature group of sequence complexity was found to be the important complementary features. From the ranking of Figure 3, it is worth noting that some components of sequence complexity feature group, such as SC-1 and SC-2, are important for prediction. Let us recall the definitions of SC-1 and SC-2 features (see Materials and Methods, 600-bp window): SC-1 feature is exactly the fourth point of complexity function and SC-2 feature is exactly the fifth point of complexity function. That means that methylated samples and unmethylated samples probably have different complexities of four-nucleotide and five-nucleotide usages of DNA sequence.

**Figure 5:**
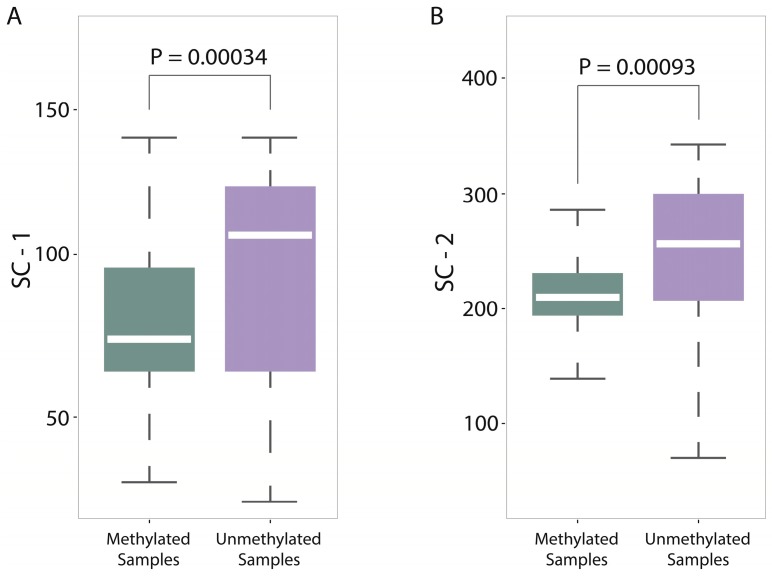
Distribution differences between methylated samples and unmethylated samples on two SC features: (**A**) box-plots of the distributions of methylated samples and unmethylated samples on SC-1 feature and corresponding statistical test (*p*-value = 0.00034); and (**B**) box-plots of the distributions of methylated samples and unmethylated samples on SC-2 feature and corresponding statistical test (*p*-value = 0.00093).

For a deep discussion, we further analyzed the distribution differences between methylated samples and unmethylated samples on SC-1 and SC-2 features (600-bp window) and then performed the corresponding statistical tests (*T* test). Both results were shown in Figure 5A,B, from which we found two interesting things: (1) The *p*-value on SC-1 feature is 0.00034 and the *p*-value on SC-2 feature is 0.00093, which shows that there are statistically significant differences between methylated samples and unmethylated ones on these two features. Therefore, it is not difficult to understand why the group of sequence complexity is an important group of features; (2) The variations of methylated samples are obviously lower than those of unmethylated samples for both two features (for SC-1 feature, variation of methylated samples equals to 60.62 and variation of unmethylated samples equals to 307.94; for SC-2 feature, variation of methylated samples equals to 90.47 and variation of unmethylated samples equals to 980.82), which implies that methylated samples tend to be more conservative with having more consistent complexity of four-nucleotide and five-nucleotide usages, whereas unmethylated samples tend to use them more randomly. That leads us to conjecture that DNA sequence motifs with length four or five are influential factors for DNA methylation, which should be validated by more future studies.

Moreover, we compared four common classifiers, and SVM resulted in the best records. We also compared two existing methods, **iDNA-Methyl** [41] and **DeepMethyl** [48] to show the advantages of our method. Additionally, we tested our model on methylation data of mouse genome for demonstrating that our model has certain generalization ability. Finally, a statistical test of experimental data and predicted data on 10 functional regions annotated by ChromHMM found that six regions were consistent, which implies reliable prediction of unassayed CpG sites.

Based on the above efforts, we summarize that our predictor brings some new benefits to the area of DNA methylation prediction:

1. Methodology: The biggest novelty of this work is the successful utilization of sequence complexity features for characterizing DNA methylation patterns. Earlier methods for selecting rational points when estimating topological entropy were not accurate and left out useful information. We provide a simple way to detect intrinsic features of sequence complexities, which were successfully used to predict DNA methylation status. Moreover, the feature importance analyses show that sequence complexities are the important complementary features.
2. Predicting window size: Previous works used different window sizes for predictions, such as 41 bp [41], 400 bp [46], and 100–1000 bp [48]. We found that 600 bp is the most appropriate by considering pairwise combinations of 22 chromosomes and different window sizes (from 100 to 1000 bp) based on a large dataset.
3. Prediction of unassayed CpG sites: A statistical test of the average methylation level of experimental CpG sites and predicted CpG sites (unassayed CpG sites, predicted by our trained model) on 10 functional regions annotated by ChromHMM found six regions were consistent. Based on this, we believe that the average methylation level of specific functional regulatory regions (such as promoters and enhancers) can be reliably predicted by our model.

The above in-depth analyses demonstrated the advantages of our computational model from different perspectives, however, there are still some limitations. For example, our predicting model only depends on the primary DNA sequence, which results in the same features extracted from different cells or cell lines. This might lead to the predicting deviation when predicting the DNA methylation level of different cells which show great changes in DNA methylation, especially between normal cells and cancer cells.

Moreover, important future work includes the investigation of the regulatory roles of DNA methylation, including the relationships between DNA methylation and transcription factor binding, especially in important regions of DNA regulatory elements, such as promoters and enhancers. Another future work is to establish a web-server to make our predictor publicly available just as shown in many recent publications (see, e.g., [52,53,55,57,63,64,65,66,67,68,69]) and to significantly enhance the influence of our predictor.

## 4. Materials and Methods

### 4.1. Features for Prediction

For our computational approach, each DNA fragment was represented as a numerical vector for inputting into the SVM for classification. In this work, the following two groups of features were used for formulating DNA fragments:

#### 4.1.1. DNA Composition (DC, 65 Features)

1. Trinucleotide frequency (TriFre, 64 features)

Trinucleotide frequency is the simplest way to formulate DNA sequences. Precisely, a DNA sequence *ω* with L bases is denoted as:

(1) $$\omega =R_{1}R_{2}R_{3}R_{4}R_{5}\cdots R_{L}$$

Trinucleotide frequency of *ω* is defined as the normalized frequency of each trinucleotide in *ω*; i.e.,

(2) $$TriFre={\left[f_{1},f_{2},f_{3,}\cdots ,f_{64}\right]}^{T}$$

where $f_{i}=\frac{n_{i}}{L-2}$, and $n_{i}$ is to count the number of the *i*-th trinucleotide occurred in *ω*. Specifically, $f_{1}=f(AAA)=\frac{n_{1}}{L-2}$, $f_{2}=f(AAA)=\frac{n_{2}}{L-2}$ and $n_{1}$, $n_{2}$, are the occurrence number of AAA and AAC in *ω*, respectively.

2. GC content (GC, 1 features)

A number of previous studies all found that GC content was a predictive feature for methylation status [34,40,46], and here we also employ GC content as a feature. The detailed computational formula of GC content is simple:

(3) $$GC=\frac{G+c}{L}$$

#### 4.1.2. Sequence Complexity (SC, 7 Features)

The concept of sequence complexity originally came from the research area of “combinatorics on words”, and it had wide applications in natural language processing, pattern matching and coding theory [70]. In general, it studies combinatorial features of sequence by investigating factor complexity, and complexity function and topological entropy are two important research topics in this area [60,71,72,73,74,75]. More precisely, to study DNA sequence, here we restricted attentions on a four-letter alphabet Ω={A,C,G,T}. Given a DNA sequence ω over Ω with finite length |ω|, its complexity function is defined as:

**Definition** **1.** *(Complexity Function, CF [70,71]). For a DNA sequence* ω *over* Ω*, the complexity function* $p_{\omega }:N\to N$ *is given by*

(4) $$p_{\omega }(n)=\#\{u:u≺\omega ,\left|u\right|=n\}$$

*where* # *denotes the number of elements of a set and* u≺ω *represents that* u *is a factor (or a subword) of* ω.

We show below, as an example, the complexity function of a short DNA fragment CAGATGTACA:

$$p_{\omega }(1)=4,\;p_{\omega }(2)=8,\;p_{\omega }(3)=8,\;p_{\omega }(4)=7,\;p_{\omega }(5)=6\;p_{\omega }(6)=5,\;p_{\omega }(7)=4,\;p_{\omega }(8)=3,\;p_{\omega }(9)=2,\;p_{\omega }(10)=1.$$

The complexity function of a sequence describes its combinatorial features, which imply that the more different factors the sequence uses, the larger the CF value is. Mathematically, we can find that $1\leq p_{\omega }(n)\leq 4^{n}$ for an arbitrary DNA sequence, and $p_{\omega }(n)=1$ represents a complete repeated sequence, whereas $p_{\omega }(n)=4^{n}$ indicates that the sequence contains all factors with full complexity.

**Definition** **2.** *(Topological Entropy, TE [70,71])**.** For an infinite sequence* ω *over* Ω*, the topological entropy* $H_{top}$ *is defined by*

(5) $$H_{top}(\omega )=\underset{n\to \infty }{\lim}\frac{\log_{4}p_{w}(n)}{n}$$

By the mathematical definition of TE, it is a quantitative index for describing the exponential increasing speed of CF. That is, if $H_{top}=c$, $p_{\omega }(n)\approx 4^{cn}$. An early related work found that randomly generated sequences had larger TE than DNA sequences, which means that DNA sequences are not randomly evolved and have certain conserved features [72]. Subsequently, a number of previous works used TE to quantitatively study the different regions across the human genome, which included promoters, exons and introns [71,72]. They found that intron > exon > promoter when considering TE [71,72]. These remarkable progresses motivated us to use CF and TE as mathematical tools to study pattern of methylation sites.

**Figure 6:**
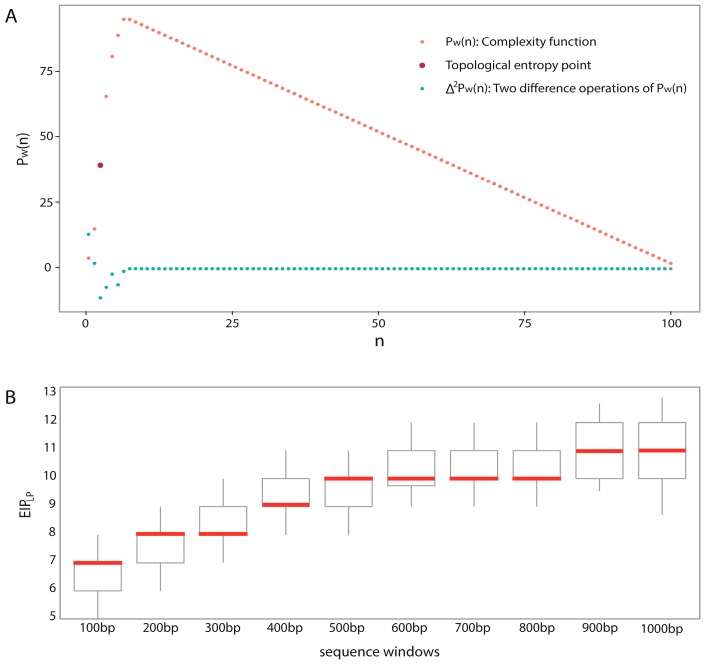
The determination approach of the exponentially increasing part of the complexity function graph: (**A**) The complexity function graph of 100-bp window DNA fragment with a methylated CpG site in the center. The red point represents the corresponding point of complexity function, the dark red point represents the point of the topological entropy point, and the blue point represents the corresponding point of two difference operations of complexity function; (**B**) The box plots of the distributions of EIP_LP_ for different window sizes.

However, the computational algorithm of TE is not easy because the mathematical definition of TE is based on an infinite sequence. For improved understanding, we generated a complexity function graph (Figure 6A) for an example, and it was observed that CF increased rapidly at the first 7 points and subsequently decreased slowly. Recall that TE represents the exponential increasing speed of CF, so we only need to focus on the first seven points. Importantly, what points should we choose to estimate TE? In 2011, Koslicki [71] provided an answer by choosing the rational point $n_{0}$ as follows:

(6) $$4^{n_{0}}+n_{0}-1<\left|w\right|\leq 4^{n_{0}+1}+(n_{0}+1)-1$$

For our 100-bp DNA sequence, easy computation would determine $n_{0}$ as 3, which was marked in Figure 6A. Later, Jin et al. [60] proposed that other points also provided complexity information besides $n_{0}$, which should be considered together to give a more precise estimation of TE in 2014. They concluded that TE should be computed as:

(7) $$H_{top}(\omega )=\frac{1}{k}\sum_{i=n_{0}-k}^{n_{0}}\frac{\log_{4}p_{\omega }(i)}{i}$$

which implied that *k* preceding points of $n_{0}$ are brought together to computation (the authors used *k* = 3 in their paper).

In this paper, we developed a novel method to choose rational points of CF to be predictive features for DNA methylation. This was based on the original definition of TE, which is represented as the exponentially increasing speed of CF. Thus, it is significant to figure out the exponentially increasing part of the graph of CF. For this purpose, the difference operations were employed here to determine the exponentially increasing part:

(8) $$\Delta p_{\omega }(k)=p_{\omega }(k+1)-p_{\omega }(k)，k=1,\ldots ,L-1$$

If successive points show non-zero patterns after several difference operations, they are probably the exponentially increasing part. More precisely, after two difference operations on complexity function, most of points were zero except for consecutive seven points, i.e., the first point to the seventh point (Figure 6A). By this way, seven points (1st–7th) were determined and were considered as the exponentially increasing part of this DNA sequence. Interestingly, these seven points not only contained $n_{0}$ which was considered as the topological entropy point, but also contained the three preceding points of $n_{0}$ (1st–3rd) which were proven to be important by Jin et al. [60]. This implies that our method of choosing rational points provide more information beyond two previous works.

Next, for investigating whether other DNA sequences of 100 bp all have a similar pattern, we performed the same strategy on 100 different DNA sequences and recorded the Last Point of the Exponentially Increasing Part ${\mathrm{EIP}}_{\mathrm{LP}}$ of each DNA sequence, and the distribution of ${\mathrm{EIP}}_{\mathrm{LP}}$ were shown by box plot in Figure 6B with median of 7. That means, most sequences have their exponentially increasing part from the 1st to 7th points, and the sequence complexity features were computed as:

(9) $$SC_{100}={\left[p_{\omega }(1),\cdots ,p_{\omega }(7)\right]}^{T}$$

We applied the above procedure to different window sizes, and the corresponding ${\mathrm{EIP}}_{\mathrm{LP}}$ were shown by box plots in Figure 6B. For example, the sequence complexity features of 600-bp DNA sequence were computed as:

(10) $$SC_{600}={\left[p_{\omega }(4),\cdots ,p_{\omega }(10)\right]}^{T}$$

For more details, the exact interval for each window size was shown in Table 8. For showing the superiority of our method，we compared three methods—entropy point (1 dim), three preceding points of entropy point (3 dim), our rational points (7 dim) by ACC index (see Table S4).

### 4.2. Support Vector Machine

Support vector machine is a common-used machine-learning algorithm for classification or regression tasks. Because of its powerful advantages on easy implementation (only two parameters needed to determine) and small samples, it became one of the most successful machine-learning tools in the last two decades. Actually, SVM maps the input (feature vector) from Euclidean space into a higher dimensional Hilbert space by a suitable kernel function. Then, it searches the Optimal Separating Hyper plane (OSH) to separate the positive samples and negative samples with the best accuracy by optimizing a given objective function. Comprehensive theory and its wide applications of SVM can be found in a famous monograph written by Vapnik [76]. In this research, we adopted a popular tool called “LibSVM 3.17” (open source, and can be downloaded from: http://www.csie.ntu.edu.tw/~cjlin/libsvm/index.html) to implement SVM with the linear kernel function.

### 4.3. Evaluating Indicator

Generally, the following three criteria are often used to evaluate a predictor for its prediction ability: independent testing, subsampling (K-fold cross-validation) test and jackknife test [77]. In this paper, we chose 10-fold cross-validation due to the new large dataset for all chromosomes and adopted independent testing for subsequent analyses. Usually, performance of a prediction method is measured by sensitivity (Sens), specificity (Spec), accuracy (ACC) and Matthew’s correlation coefficient (MCC) value, calculated as:

(11) $$\left\{ \begin{array}{l} \mathrm{Sens}=\frac{\mathrm{TP}}{\mathrm{TP}+\mathrm{FN}}\\ \mathrm{Spec}=\frac{\mathrm{TN}}{\mathrm{TN}+\mathrm{FP}}\\ \mathrm{ACC}=\frac{\mathrm{TP}+\mathrm{TN}}{\mathrm{TP}+\mathrm{FP}+\mathrm{TN}+\mathrm{FN}}\\ \mathrm{MCC}=\frac{\mathrm{TP}\times \mathrm{TN}\;-\;\mathrm{FP}\times \mathrm{FN}}{\sqrt{\left(\mathrm{TP}+\mathrm{FN})(\mathrm{TP}+\mathrm{FP})(\mathrm{TN}+\mathrm{FP})(\mathrm{TN}+\mathrm{FN}\right)}} \end{array}\right.$$

where TP represents the number of true positives (methylated CpG sites predicted as methylated CpG sites) in one experiment, and TN represents the number of true negatives (unmethylated CpG sites predicted as unmethylated CpG sites). Similarly, FP represents the number of false positives (methylated CpG sites predicted as unmethylated CpG sites), and FN represents the number of false negatives (unmethylated CpG sites predicted as methylated CpG sites). Based on these four terms, we use the term “ACC” to represent the prediction accuracy of our model using Equation (11). For more intuitive and easier to understand these formulations, please refer to some recent publications [63,64,65,66,67,78,79]. Moreover, the above metrics is valid only for the single-label systems. For the multi-label systems whose existence has become more frequent in system biology and system medicine [56,80,81,82]. Additionally, to test the balance between true positive and false positive rates of a predictor, another important evaluating indicator is the Area Under the ROC Curve (AUC). The predictor is considered as a better predictor when the AUC value is larger.

## Figures and Tables

**Table 1:** Detailed information on the four datasets used in this work. The cutoff threshold of CD-HIT was 0.8.

Species or Cell Lines	Number of Positive Samples	Number of Positive Samples after CD-HIT	Number of Negative Samples	Number of Negative Samples after CD-HIT	Data Resource (GEO Number)
Homo sapiens	139,931	104,876	536,183	159,090	GSE49828
Mouse	190,000	135,733	190,000	138,207	GSE56697
GM12878	263,698	166,116	563,702	252,188	GSM683906
K562	292,791	159,310	540,465	251,948	GSM683856

**Table 2:** Comparison of four commonly used classifiers using independent testing on IT-set. MCC is the abbreviation of Matthew’s Coefficient Of Correlation; Sens is the abbreviation of sensitivity; Spec is the abbreviation of specificity.

Classifiers	Dimension	ACC	AUC	MCC	Sens	Spec
Support Vector Machine	72	0.971	0.996	0.940	0.972	0.969
Random Forest	72	0.959	0.984	0.917	0.958	0.959
NaiveBayes	72	0.881	0.937	0.766	0.927	0.844
K-Nearest Neighbor	72	0.849	0.941	0.706	0.803	0.911

**Table 3:** Comparison of two groups of features using independent testing on IT-set.

Feature Set	Dimension	ACC	AUC	MCC	Sens	Spec
All 72 Features	72	0.971	0.996	0.940	0.972	0.969
DNA Composition	65	0.946	0.990	0.892	0.953	0.939
Sequence complexity	7	0.910	0.968	0.819	0.911	0.909

**Table 4:** Comparison of top 24 important features and remaining 48 features using independent testing on IT-set.

Feature Set	Dimension	ACC	AUC	MCC	Sens	Spec
All 72 Features	72	0.971	0.996	0.940	0.972	0.969
Top 24 Important Features	24	0.958	0.990	0.915	0.956	0.959
Other 48 Features	48	0.907	0.969	0.815	0.910	0.905

**Table 5:** Comparison with **iDNA-Methyl**.

Predictor	ACC	MCC	Sens	Spec
**iDNA-Methyl**	77.49	54.71	61.25	90.33
Our work	78.62	57.23	78.68	78.56

**Table 6:** Comparison with **DeepMethyl**.

Cell Line	Predictor	Chromosome	Window Size	ACC	MCC	Sens	Spec
GM12878	**DeepMethyl**	Chr1	500	0.900	0.800	0.905	0.894
GM12878	Our method	Chr1	600	0.984	0.969	0.985	0.984
GM12878	**DeepMethyl**	Chr21	600	0.942	0.886	0.966	0.918
GM12878	Our method	Chr21	600	0.983	0.966	0.985	0.981
K562	**DeepMethyl**	Chr1	600	0.823	0.649	0.784	0.863
K562	Our method	Chr1	600	0.976	0.952	0.975	0.978
K562	**DeepMethyl**	Chr21	800	0.876	0.753	0.904	0.848
K562	Our method	Chr21	600	0.979	0.958	0.974	0.985

**Table 7:** Prediction results of three mouse chromosomes using our method.

Chromosome	Dimension	ACC	AUC	MCC	Sens	Spec
Mouse chromosome 1	72	0.980	0.998	0.961	0.984	0.977
Mouse chromosome 10	72	0.982	0.998	0.964	0.978	0.986
Mouse chromosome 19	72	0.940	0.985	0.879	0.926	0.953

**Table 8:** The determinations of seven sequence complexity features of different window sizes. |ω| represents the length of window, and *n*_0_ represents the topological entropy point in reference [35].

|ω|	*n*_0_	Sequence Complexity Features (SC Features)	EIP_LP_
100 bp	3	1–7	7
200 bp	3	2–8	8
300 bp	4	2–8	8
400 bp	4	3–9	9
500 bp	4	4–10	10
600 bp	5	4–10	10
700 bp	5	4–10	10
800 bp	5	4–10	10
900 bp	5	5–11	11

## Supplementary Materials

Supplementary materials can be found at www.mdpi.com/1422-0067/18/2/420/s1.
