# Supplementary material for: Genome-Wide Prediction of DNA Methylation Using DNA Composition and Sequence Complexity in Human
Source: Int J Mol Sci. 2017 Feb 16;18(2):420. doi: 10.3390/ijms18020420 (PMC5343954; doi:10.3390/ijms18020420)
Supplement: Supplementary file 1 [file ijms-18-00420-s001.pdf]

Supplementary Materials: Genome-Wide Prediction of DNA Methylation Using DNA Composition and Sequence Complexity in Human

Chengchao Wu, Shixin Yao Xinghao Li Chujia Chen and Xuehai Hu

Table S1. The prediction accuracies for combinations of different chromosomes and different window sizes. The values are ACC and the values with red color are the maximums within the same chromosomes. Nine out of all 22 chromosomes achieve its maximal prediction results in 600 bp-windows. The values in every grid represent the prediction accuracies (ACC).

**Chromesome**	**100 bp**	**200 bp**	**300 bp**	**400 bp**	**500 bp**	**600 bp**	**700 bp**	**800 bp**	**900 bp**	**1000 bp**
Chr1	0.9484	0.9527	0.9707	0.9701	0.9724	0.9703	0.9741	0.9718	0.9675	0.9752
Chr2	0.9479	0.941	0.9576	0.9665	0.9665	0.9688	0.9602	0.9642	0.9599	0.9596
Chr3	0.9379	0.9402	0.9523	0.9652	0.9609	0.9641	0.9531	0.9582	0.9594	0.9594
Chr4	0.9133	0.9211	0.9352	0.9451	0.9404	0.9425	0.9472	0.9566	0.9551	0.9514
Chr5	0.916	0.9291	0.9423	0.9532	0.9514	0.9501	0.9554	0.9532	0.9528	0.9541
Chr6	0.934	0.9304	0.947	0.9462	0.949	0.9518	0.9514	0.9502	0.9478	0.9486
Chr7	0.9205	0.9209	0.9428	0.9501	0.9538	0.944	0.9448	0.9538	0.9534	0.957
Chr8	0.895	0.9127	0.9304	0.9501	0.9496	0.9584	0.9522	0.9439	0.9402	0.933
Chr9	0.9273	0.9255	0.9436	0.9562	0.9486	0.958	0.9458	0.9495	0.9508	0.9513
Chr10	0.918	0.9115	0.924	0.9336	0.9497	0.9557	0.9431	0.9497	0.9457	0.9484
Chr11	0.9132	0.9193	0.9507	0.9526	0.9595	0.9503	0.9614	0.9545	0.948	0.9576
Chr12	0.9249	0.9323	0.9463	0.9537	0.9533	0.9507	0.9498	0.9515	0.9384	0.9472
Chr13	0.8835	0.8835	0.9097	0.9252	0.9097	0.9262	0.932	0.9146	0.9359	0.9223
Chr14	0.9298	0.9232	0.9364	0.937	0.9448	0.9496	0.9418	0.9634	0.9436	0.9484
Chr15	0.9051	0.9223	0.9455	0.9419	0.9486	0.9468	0.9406	0.9339	0.9498	0.9364
Chr16	0.8893	0.9112	0.9318	0.9444	0.9471	0.9545	0.9484	0.9475	0.9444	0.9462
Chr17	0.925	0.9326	0.9452	0.9576	0.9617	0.9658	0.9576	0.9592	0.949	0.9455
Chr18	0.8895	0.8843	0.9215	0.9112	0.9246	0.9236	0.9225	0.9153	0.9174	0.9246
Chr19	0.9123	0.9282	0.9349	0.9579	0.9471	0.9535	0.9436	0.946	0.937	0.9425
Chr20	0.8908	0.8992	0.9231	0.9264	0.9373	0.9328	0.9276	0.9335	0.9302	0.9348
Chr21	0.788	0.7989	0.8678	0.8841	0.8732	0.8786	0.8641	0.8804	0.9022	0.8931
Chr22	0.8727	0.8975	0.9161	0.9177	0.9255	0.9379	0.9286	0.9099	0.934	0.9278
Average	0.9083	0.9144	0.9352	0.9430	0.9443	0.9470	0.9430	0.9437	0.9438	0.9438

Table S2. The prediction results for two cell lines, GM12878 and K562. DeepMethyl only listed limited prediction results in their paper [29], and here we list all the prediction results of all chromosomes using 600 bp window size. The values in every grid represent the prediction accuracies (ACC).

Chromosome	GM12878		K562	
	Our Work	DeepMethyl	Our Work	DeepMethyl
Chr1	0.984	0.900	0.976	0.823
Chr2	0.983	—	0.971	—
Chr3	0.983	—	0.968	—
Chr4	0.986	—	0.985	—
Chr5	0.979	—	0.977	—
Chr6	0.958	—	0.984	—
Chr7	0.973	—	0.978	—
Chr8	0.976	—	0.974	—
Chr9	0.988	—	0.987	—
Chr10	0.963	—	0.971	—
Chr11	0.966	—	0.962	—
Chr12	0.973	—	0.956	—
Chr13	0.988	—	0.969	—
Chr14	0.980	—	0.971	—
Chr15	0.987	—	0.973	—
Chr16	0.981	—	0.962	—
Chr17	0.987	—	0.968	—
Chr18	0.987	—	0.959	—
Chr19	0.969	—	0.968	—
Chr20	0.985	—	0.969	—
Chr21	0.983	0.942	0.979	0.876
Chr22	0.985	–	0.973	–

Table S3. The results for genome-wide mouse DNA methylation prediction. The meanings of ACC, AUC, MCC, Sens, and Spec can be found in "Materials and Methods".

Chromosome	ACC	AUC	MCC	Sens	Spec
Chr1	0.9803	0.9980	0.9606	0.9841	0.9765
Chr2	0.9786	0.9962	0.9572	0.9733	0.9839
Chr3	0.9787	0.9977	0.9574	0.9767	0.9807
Chr4	0.9772	0.9976	0.9543	0.9796	0.9747
Chr5	0.9808	0.9973	0.9616	0.9796	0.9820
Chr6	0.9801	0.9951	0.9604	0.9717	0.9888
Chr7	0.9685	0.9931	0.9369	0.9670	0.9699
Chr8	0.9838	0.9978	0.9676	0.9825	0.9851
Chr9	0.9739	0.9939	0.9480	0.9654	0.9828
Chr10	0.9821	0.9980	0.9642	0.9781	0.9860
Chr11	0.9730	0.9958	0.9460	0.9704	0.9757
Chr12	0.9762	0.9942	0.9525	0.9711	0.9814
Chr13	0.9783	0.9977	0.9565	0.9769	0.9797
Chr14	0.9592	0.9936	0.9185	0.9494	0.9693
Chr15	0.9717	0.9971	0.9436	0.9619	0.9819
Chr16	0.9695	0.9945	0.9393	0.9596	0.9799
Chr17	0.9771	0.9962	0.9542	0.9710	0.9833
Chr18	0.9717	0.9942	0.9436	0.9654	0.9783
Chr19	0.9395	0.9857	0.8791	0.9264	0.9531
Average	0.9737	0.9955	0.9475	0.9690	0.9786

To show the superiority of our method (600 bp as an example), ten groups of samples were chosen from our large data set with 5000 positive and 5000 negative samples in each group (50,000 + 50,000 in total), and each group was evaluated by three methods—entropy point (1 dim), three preceding points of entropy point (3 dim), our rational points (7 dim) by ACC index and 10-fold cross-validation (see Table S4). Finally, two Wilcoxon rank tests were performed between two previous methods and our method based on the ten groups of results, and significant increases were found in the statistical tests (see Table S4, p values are all 9.03×10^{-5}), which show that our method with rationally choosing points is superior to existing methods.

Table S4. The comparative analysis of two previous works and our method for choosing rational sequence complexity features.

Group	Topological Entropy [68], 1 dim	Our Method, 7 dim	Jin et al. [57], 3 dim
1	0.5756	0.8950	0.8494
2	0.5842	0.8910	0.8514
3	0.5768	0.8926	0.8516
4	0.5668	0.8920	0.8536
5	0.5660	0.8976	0.8440
6	0.5676	0.8918	0.8410
7	0.5668	0.8944	0.8520
8	0.5852	0.8914	0.8530
9	0.5612	0.8926	0.8448
10	0.5680	0.8884	0.8536
p value, Wilcoxon test	9.03×10^{-5}		9.03×10^{-5}
p value, T test	2.2×10^{-16}		2.3×10^{-13}
